# The Darwin cure for apiculture? Natural selection and managed honeybee health

**DOI:** 10.1111/eva.12448

**Published:** 2016-12-26

**Authors:** Peter Neumann, Tjeerd Blacquière

**Affiliations:** ^1^ Institute of Bee HealthVetsuisse FacultyUniversity of BernBernSwitzerland; ^2^ Bees@wurBio‐interactions and Plant HealthWageningen URWageningenThe Netherlands

**Keywords:** *Apis mellifera*, beekeeping, honeybee, natural selection

## Abstract

Recent major losses of managed honeybee, *Apis mellifera,* colonies at a global scale have resulted in a multitude of research efforts to identify the underlying mechanisms. Numerous factors acting singly and/or in combination have been identified, ranging from pathogens, over nutrition to pesticides. However, the role of apiculture in limiting natural selection has largely been ignored. This is unfortunate, because honeybees are more exposed to environmental stressors compared to other livestock and management can severely compromise bee health. Here, we briefly review apicultural factors that influence bee health and focus on those most likely interfering with natural selection, which offers a broad range of evolutionary applications for field practice. Despite intense breeding over centuries, natural selection appears to be much more relevant for the health of managed *A. mellifera* colonies than previously thought. We conclude that sustainable solutions for the apicultural sector can only be achieved by taking advantage of natural selection and not by attempting to limit it.

## 1 Introduction

**Figure 1:**
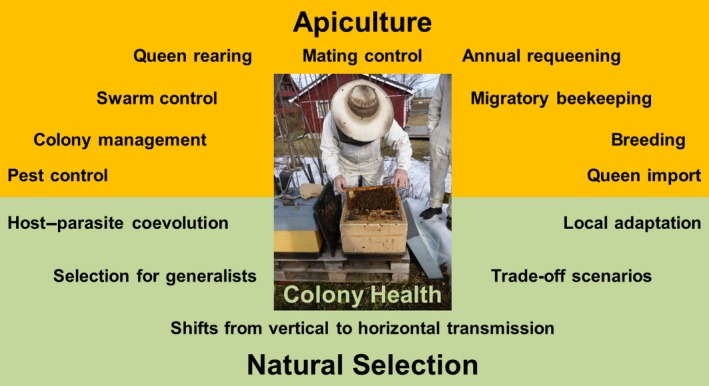
Apiculture and natural selection as a joint framework for the health of managed honeybee colonies. Specific beekeeping methods, which are likely to interfere with natural selection (=orange area), and possible impact on natural selection (=green area) are shown with an ongoing colony inspection in the center

The western honeybee, *Apis mellifera,* is one of the most economically important insects, providing essential pollination services for human food security as well as valuable hive products for the apicultural sector (Klein et al., 2007; Morse & Calderone, 2000). Therefore, major losses of managed *A. mellifera* colonies at a global scale (e.g., van Engelsdorp & Meixner, 2010; vanEngelsdorp, Hayes Jr., Underwood, Caron, & Pettis 2011; Neumann & Carreck, 2010; Pirk, Human, Crewe, & vanEngelsdorp, 2014) have resulted in a multitude of national and international research efforts to identify underlying mechanisms (Moritz et al., 2010; Potts et al., 2011; Vanbergen et al., 2012; among many others). Numerous factors acting singly and/or in combination have been identified, ranging from pathogens, over nutrition to pesticides (see Potts et al., 2010 for an overview). However, the role of apiculture as another stressor has received far less attention, although management can severely compromise bee health. In particular, the role of common beekeeping practices in limiting natural selection as a potential major factor governing managed honeybee health has been completely ignored so far. This is kind of surprising, because it is well known that honeybees are more exposed to environmental stressors compared to other livestock. As natural selection is the key mechanism of evolution, it will enable any given stock of managed honeybees, irrespective of habitat (agro‐ecosystems, nature reserves, etc.) and/or genetic background (endemic, imported, “pure” breeding lines, hybrids [e.g., Buckfast], etc.) to adapt to each and every stressor as long as the ability to cope with the stressor has a genetic basis so that the respective heritable traits can change in this population over time. Although domestication always interferes by definition with natural selection and apicultural selection has existed for decades, if not centuries (Crane, 1999), we here argue that beekeeping interference with natural selection in combination with globalization of industrialized apiculture may have now reached levels, where ill effects are inevitable at the colony level. Such ill effects have previously and repeatedly been reported in populations of managed honeybees (see review by van Engelsdorp & Meixner, 2010), but the role of natural selection has not been considered in this regard. Even though comparisons with historical data sets remain notoriously difficult, it appears as if the factors compromising managed honeybee health may have reached higher levels compared to the past (invasive pests, vectored viruses, prophylactic pesticide usage, starvation, etc., reviewed by Potts et al., 2010). Indeed, globally standardized survey data from the COLOSS network over the past 8 years (www.coloss.org) suggest unsustainable high losses repeatedly in many regions globally. Here, we therefore briefly review apicultural factors governing honeybee health and focus on those probably interfering with natural selection (Figure 1), which offers a broad range of evolutionary applications for field practice.

It is evident that the beekeeper is the most crucial (multi)factor driving managed honeybee health. Indeed, beekeepers play the key role in spread as well as diagnosis and control of new and established diseases (Mutinelli, 2011; Neumann, Pettis, & Schäfer, 2016; Rosenkranz, Aumeier, & Ziegelmann, 2010), for example, treating against ectoparasitic mites, *Varroa destructor* (Rosenkranz et al., 2010), not only prevents host–parasite coevolution, but may also add to the exposure to pesticides thereby possibly compromising colony health (Boncristiani et al., 2012). In general, the high density of colonies at apiaries promotes disease transmission and impact (Seeley & Smith, 2015) and the large hives compared to natural nests may also have a detrimental impact on colony survival (Loftus, Smith, & Seeley, 2016). During routine colony inspections, beekeepers frequently break the natural propolis envelope of colonies, which may compromise social immunity (Simone‐Finstrom, Evans, & Spivak, 2009). Apiculture also governs bee nutrition, for example, by placing stationary apiaries in areas with bad forage or by choosing the forage for the bees in migratory beekeeping. The alternation of honey/pollen flows with poor forage periods is indeed a challenge to the colonies to adapt to normal seasonality (Bretagnolle & Gaba, 2015) and may affect resilience to diseases. Replacing diverse honey stores with low‐quality sugar water may also impact health (Erler, Denner, Bobis, Forsgren, & Moritz, 2014; Wheeler & Robinson, 2014), and untimely and/or insufficient feeding of honey‐depleted colonies for overwintering is an obvious key reason for mortality (vanEngelsdorp et al., 2011). Finally, due to the potential role of endosymbionts and the entire associated microbiome of honeybees (Aebi & Neumann, 2011; Engel et al., 2016), treatment of colonies with acaricides (Kakumanu, Reeves, Anderson, Rodrigues, & Williams, 2016), antibiotics, and even sugar feeding may interfere with natural population dynamics of such associated prokaryotes. All these factors have received at least some attention for improving bee health in the past. However, the limitation of natural selection by beekeepers has so far been ignored for mitigation measures.

While treatment against disease is helpful, it nevertheless prevents natural selection for improved host resistance and tolerance (Fries & Bommarco, 2007; Råberg, Graham, & Read, 2009). In particular, the common practice of removing male sexuals (=drone brood) to control *V. destructor* (Rosenkranz et al., 2010), basically castrates colonies, thereby preventing that well‐adapted ones spread their genes in the population. This seems significant because recent evidence suggests substantial local adaptations of honeybees enhancing colony survival (Büchler et al., 2014) and reducing pathogen loads (Francis et al., 2014). In this regard, the situation in Europe is different to areas, in which European honeybees have been imported. Indeed, several local subspecies can be differentiated in Europe using morphometric or genetic makers (Miguel, Iriondo, Garnery, Sheppard, & Estonba, 2007; Miguel et al., 2011; Ruttner, 1988). The competition of introduced honeybees with such endemic honeybees and other pollinators is plausible (see Moritz, Härtel, & Neumann, 2005; for a review), but this is not a focus of this article. Indeed, we here argue about natural selection and managed honeybee health and not about conservation of endangered honeybee subspecies. Clearly, each honeybee subspecies deserves to be protected in its own rights and local adaptations are most likely (e.g., endemic *A. m. mellifera* in France, Strange, Garnery, & Sheppard, 2007). We cannot and do not want to question this obvious nature conservation issue, especially because adapted traits of endemic subspecies may be lost due to introgression of foreign ones (Meixner et al., 2010). However, the well‐justified ongoing nature conservation efforts (mainly in Europe) and our suggestion to take advantage of natural selection to improve the health of managed honeybee colonies globally are basically two different things. For a functional global apiculture, the health of any given colony seems to be more relevant than conservation efforts for specific subspecies in Europe or elsewhere. This is especially true, because there are nowadays more managed colonies of European honeybees outside of Europe than in Europe itself (FAO data: http://faostat.fao.org/). For example, susceptibility to infection by the endoparasitic microsporidian *Nosema ceranae* is not linked to honeybee taxa, but results from the variability between colonies, and those differences are probably linked to genetic variations (Fontbonne et al., 2013).

These genotype–environment interactions, including immuno‐priming of eggs by the queen in response to pathogens in the hive (Salmela, Amdam, & Freitak, 2015), are routinely and constantly disrupted when queens or colonies are moved over large distances, for example, from Southern Italy to Finland, as part of international apicultural trade. Indeed, the industrial production of tens of thousands of queens annually, which are nowadays exported at a continental and even global scale (Lodesani & Costa, 2003), clearly interferes with any local adaptations. Therefore, “think globally, but breed locally” appears an adequate suggestion for honeybee breeders to take advantage of natural selection and to foster local adaptations.

In artificial insemination, breeders choose drones (=male sexuals) of the right age, which obviously have not made it yet to drone congregation areas and may thus not have the full reproductive potential. At isolated mating apiaries, only few drone‐producing colonies are provided, which are often headed by sister queens, thereby clearly limiting the full potential of the highly polyandrous mating system of honeybees to generate subfamilies with ample genotypic diversity and respective derived benefits (Oldroyd & Fewell, 2007; Mattila & Seeley, 2007; Tarpy, vanEngelsdorp, & Pettis, 2013). The equal number of matings of wild and managed queens (Tarpy, Delaney, & Seeley, 2015) suggests that the system has evolved to provide optimal genetic variation of colonies, but will fail to deliver with closer genetic similarity of the drones and reduced mate numbers. A recent study showed that honeybee colonies, which were made hyperpolyandrous artificially (30 or 60 matings), had improved performance (Delaplane, Pietravalle, Brown, and Budge (2015), thereby suggesting that genetic diversity of *A. mellifera* has already been lost and thus drone mates may be too genetically similar by now.

The buildup of a stable host–parasite relationship is strongly favored by vertical transmission of the parasite (Fries & Camazine, 2001) and is unlikely to occur when horizontal transmission is the predominant route (Schmid‐Hempel, 2011). Indeed, shifts from vertical to horizontal transmission are known to increase pathogen virulence (Woolhouse, Haydon, & Antia, 2005). However, the common practice in commercial beekeeping in most countries to routinely requeen colonies annually or every 2 years limits the full adaptive potential of vertical transmission. After requeening, parasites are confronted not only with an entirely new queen genotype, but also with novel genotypes of the drones, the queens have mated with (assuming natural queen mating at apiaries and unrelated drone/queen sources). This may have caused shifts from vertical to horizontal transmission with respective consequences for the virulence of honeybee parasites.

Commercial breeders select against swarming, defensive behavior, and propolis usage, thereby probably compromising colony defense and social immunity (Meunier, 2015). Indeed, in Africa, where the majority of honeybee colonies are not kept by man and where beekeepers are mostly side users not interfering with natural swarming, queen rearing etc., the virtually nonbred local subspecies have less desirable beekeeping traits, but a superior health compared to European ones (Pirk, Strauss, Yusuf, Démares, & Human, 2016). This supports the notion of a trade‐off scenario between commercially desired traits and bee health. In particular, queen failure is one of the foremost mentioned causes of honeybee losses (vanEngelsdorp et al., 2011; Pettis, Rice, Joselow, vanEngelsdorp, & Chaimanee, 2016) and may also be linked to breeding, because queen breeders usually ignore choices made by colonies and choose larvae based on right age alone. The natural reproductive cycle of a colony, incl. hormonal and nutritional aspects, determines timing and development of drones and new queens and often lays outside of the time window for commercial queen rearing. Moreover, during emergency queen rearing, the choice of the bees is not at random; instead, subfamilies, which are rare in the work force, are significantly more likely to end up as queens (Moritz et al., 2010). As such royal subfamilies are rare, human choice of larvae based on appropriate age alone is likely to miss those and instead offers only suboptimal choices for the bees. Moreover, breeding for *V. destructor*‐resistance over >20 years has still not resulted in survival of untreated colonies, but natural selection has delivered multiple times (Locke, 2016; Rosenkranz et al., 2010), thereby suggesting that breeders should choose traits favored by natural selection. This suggests fundamental conceptual flaws in both commercial honeybee queen rearing and breeding. As the fitness of a honeybee colony clearly is the number of surviving swarms as well as the number of successfully mating drones (all other traits are only tokens of fitness), the selection by beekeepers for low swarming tendency of colonies and removal of drone brood, mainly to combat mites *V. destructor*, remain probably the key factors in limiting natural selection.

There is amplitude of hypothesis‐driven research avenues to test our claims. For example, the possible role of suboptimal choices made by queen breeders for the recent queen‐related problems (vanEngelsdorp et al., 2011; Pettis et al., 2016) could be investigated by comparing the performance of honeybee queens natural chosen by the bees themselves with grafted ones in populations, which still have ample genetic diversity (e.g., in Africa). Similarly, given that natural selection plays the key role for survival of otherwise deadly *V. destructor* mite infestations, the famous “Bond experiment” (Locke & Fries, 2011) conducted in other countries should almost always result in at least some surviving colonies.

## 2 Conclusions

It is obvious that taking into account natural selection will not solve all of the various problems for apiculture, but instead we consider it to be a main issue in itself at the moment. As natural selection is the differential survival and reproduction of individuals due to differences in phenotype, future efforts to enhance managed honeybee health should take into account the central role of apiculture in limiting natural selection and compromising colony health via adjusted keeping and breeding of local bees. Here lies a great opportunity for beekeeping in several countries, where economic constraints are no longer leading as beekeeping has become a hobby sector, with dispersed and small apiaries being the rule. Sustainable solutions for the apicultural sector can only be achieved by taking advantage of natural selection and not by attempting to limit it.

## Data Archiving Statement

We will not be archiving data because this manuscript does not have associated data.
